# Development of CDX-527: a bispecific antibody combining PD-1 blockade and CD27 costimulation for cancer immunotherapy

**DOI:** 10.1007/s00262-020-02610-y

**Published:** 2020-05-25

**Authors:** Laura A. Vitale, Li-Zhen He, Lawrence J. Thomas, Anna Wasiuk, Thomas O’Neill, Jenifer Widger, Andrea Crocker, Laura Mills-Chen, Eric Forsberg, Jeffrey Weidlick, Colleen Patterson, Russell A. Hammond, James Boyer, Crystal Sisson, Diego Alvarado, Joel Goldstein, Henry C. Marsh, Tibor Keler

**Affiliations:** 1 Celldex Therapeutics, Inc., 53 Frontage Road, Suite 220, Hampton, NJ 08827 USA; 2 Celldex Therapeutics, Inc., 151 Martine Street, Fall River, MA 02723 USA; 3 Celldex Therapeutics, Inc., 300 George Street, Suite 530, New Haven, CT 06511 USA

**Keywords:** CD27, PD-L1, Bispecific antibody, Immunotherapy, Antigen presenting cells

## Abstract

CD27 is a costimulatory molecule that provides a complementary target to the PD-1/PD-L1 checkpoint axis on T cells. Combining a CD27 agonist antibody with PD-1/PD-L1 blockade has shown synergistic antitumor activity in preclinical models, which led to clinical studies of the combination in cancer patients. We theorized that coupling CD27 costimulation with PD-1/PD-L1 blockade in a bispecific antibody (BsAb) may provide greater immune activating properties than combining the individual mAbs due to enhanced CD27 activation by cross-linking through PD-L1 and Fc receptors. To test this approach, we developed CDX-527, a tetravalent PD-L1xCD27 IgG1-scFv BsAb. CDX-527 potently inhibits PD-1 signaling and induces CD27-mediated T cell costimulation through PD-L1 cross-linking. In mixed lymphocyte reaction assays, CDX-527 is more potent than the combination of the parental antibodies, suggesting that cross-linking through both Fc receptors and PD-L1 results in enhanced CD27 agonist activity. CDX-527 was shown to mediate effector function against tumor cells overexpressing either CD27 or PD-L1. In human CD27 transgenic mice, we observed that antigen-specific T cell responses to a vaccine are greatly enhanced with a surrogate PD-L1xCD27 BsAb. Furthermore, the BsAb exhibits greater antitumor activity than the combination of the parental antibodies in a syngeneic lymphoma model. A pilot study of CDX-527 in cynomolgus macaques confirmed a mAb-like pharmacokinetic profile without noted toxicities. These studies demonstrate that CDX-527 effectively combines PD-1 blockade and CD27 costimulation into one molecule that is more potent than combination of the parental antibodies providing the rationale to advance this BsAb toward clinical studies in cancer patients.

**Electronic supplementary material:**

The online version of this article (10.1007/s00262-020-02610-y) contains supplementary material, which is available to authorized users.

## Introduction

Antibody blockade of PD-1/PD-L1 interactions has resulted in regulatory approvals in at least 14 different cancer indications, demonstrating the broad utility of this approach [1, 2]. However, most patients do not achieve long-term responses, and additional interventions are required. Combination with targeted and chemotherapies has improved outcomes to PD-1 blockade as evidenced by the recent combination approvals in renal cell carcinoma [3] and breast cancer [4].

A significant factor in the resistance to PD-1 blockade therapy is the lack of tumor-specific T cell responses, which may be attributed to low levels of neoantigens, inefficient antigen processing and presentation and poor T cell priming [5, 6]. Therefore, combination with agents that enhance these processes is rational for improving outcomes to PD-1 blockade.

The TNFR superfamily member CD27 is a T cell costimulatory molecule with an important and non-redundant role in their activation, proliferation and survival [7–10]. This point is best illustrated in individuals with deficiency of either CD27 or its ligand CD70 that results in sometimes fatal EBV-driven lymphoproliferation, hypogammaglobulinemia and lymphoma development [11–15]. Among the costimulatory molecules 4-1BB (CD137), OX40 (CD134) and ICOS (CD278), CD27 is uniquely expressed at high levels on naïve T cells making it especially well-suited to help prime and promote new T cell responses [7, 16–18].

The potent activity of CD27 agonist antibodies in T cell activation and antitumor activity has been well documented in mouse models and human primary cultures [19, 20]. The antitumor activity of the anti-human CD27 agonist mAb, varlilumab, was established using human CD27 transgenic (huCD27-Tg) mice and found to be mediated through a combination of T cell costimulation and reduction/inhibition of Treg cells [21]. Importantly, a strong synergy in antitumor activity was observed when combining CD27 agonist mAbs with PD-1/PD-L1 blockade. Transcriptome analysis of the expanding CD8 T cells from these studies demonstrated complementary profiles of proliferation and cytotoxicity for CD27 agonism and PD-1/PD-L1 blockade, respectively, which converged in the combination treatment to yield a particularly notable enhanced expression of genes associated with T cell activation [22].

Varlilumab has been in clinical trials both as monotherapy and in combination with PD-1 blockade. As monotherapy in patients with advanced cancers, varlilumab was well tolerated at all dose levels, induced pharmacological changes consistent with the preclinical data (T cell activation and Treg depletion) and demonstrated clinical activity with 3 patients having long-term clinical benefit without any additional therapy [23]. Varlilumab in combination with nivolumab in advanced solid tumor patients was generally well tolerated through the highest dose of varlilumab (10 mg/kg) and nivolumab (3 mg/kg) administered every 2 weeks [24]. Clinical benefit was observed in patients with low probability to respond to nivolumab monotherapy. In particular, we observed durable responses in ovarian cancer patients with PD-L1 low or negative tumors and glioblastoma multiform patients with unmethylated gene promoter for O-6-methylguanine-DNA methyltransferase [25].

The strong rationale for combining CD27 costimulation and PD-1 blockade, together with the supportive preclinical and clinical data motivated us to engineer these activities into a single molecule. To this end, we developed novel CD27 and PD-L1 human antibodies using human Ig transgenic mice and expressed them as a whole IgG genetically fused to a single-chain variable fragment (scFv) yielding a tetravalent bispecific antibody (BsAb) [26] referred to as CDX-527. Here we report the generation, characterization and functional activities of CDX-527, and of a surrogate BsAb that is cross-reactive with mouse PD-L1 and used to demonstrate in vivo activity.

## Materials and methods

### Development and characterization of bispecific antibodies

Antibodies to CD27 and PD-L1 were generated by immunization of H2L2 human Ig transgenic mice (Harbour Antibodies BV) with recombinant human CD27 or PD-L1. Splenocytes were used for hybridoma preparation by standard polyethylene glycol fusion techniques. The variable heavy and light chain regions of selected antibodies were cloned into a human IgG1κ expression vector, expressed in ExpiCHO cells (Invitrogen), and further characterized. The PD-L1 antagonist mAb avelumab (AVE) was similarly prepared using the published sequence (WHO Drug Information, Vol. 29, No. 2, p. 203, 2015).

For the BsAb, an expression vector encoded the full-length anti-PD-L1 mAb 9H9 IgG1κ heavy and light chains and the scFv of the anti-CD27 2B3 mAb genetically linked in *V*_L_–*V*_H_ orientation to the C-terminus of the 9H9 mAb heavy chain. Cysteine residues were introduced, one in 2B3 *V*_L_ and one in 2B3 *V*_H_, to stabilize the scFv domains. An analogous vector was prepared in which the 9H9 *V*_H_ and *V*_L_ were replaced by the avelumab *V*_H_ and *V*_L_. These vectors were transfected into HD-BIOP3 cells (Horizon Discovery) and proteins were purified by protein A and size-exclusion chromatography. All purified mAbs and BsAbs contained < 0.5 endotoxin units/mg.

### Binding and blocking assays

#### Affinity determination using bio-layer interferometry

The mAbs or BsAbs were captured on anti-human Fc capture biosensors (ForteBio). Binding was determined by exposing the loaded biosensor to human PD-L1-HIS (R&D Systems) or human CD27 (generated in-house). Affinity measurements were determined using twofold serial dilutions of analyte ranging from 50 to 0.195 nM. The association and dissociation curves were fitted to a 1:1 binding model using the data analysis software according to the manufacturer’s guidelines.

#### ELISA assays

Extracellular domains of human and cynomolgus CD27 were generated and purified from transient transfections using Protein L. The fusion protein of human PD-L1 and mouse Ig Fc (msFc) domain was purified from transient transfections using Protein A. The following fusion proteins were purchased: mouse PD-L1 with mouse Ig Fc domain (ACROBioSystems), and mouse, cynomolgus macaque and rat PD-L1 with human Ig Fc (R&D Systems). For ELISA, plates coated with recombinant protein were exposed to samples and binding was detected using an HRP-labeled goat-anti-human IgG (Fc-specific) antibody and developed with 3,3′,5,5′-tetramethylbenzidine substrate. For BsAb binding, wells were coated with CD27 protein. BsAb dilutions were allowed to bind before adding human or mouse PD-L1-msFc which was detected with an HRP-labeled goat anti-mouse IgG (Fc specific) antibody.

#### Flow cytometry

HEK293 cells transfected with human CD27 or PD-L1 (Crown Bioscience) were incubated with mAbs for 20 min, and the bound antibodies were detected with a phycoerythrin (PE)-labeled goat anti-human IgG Fc-specific probe (Jackson ImmunoResearch). Cell-associated fluorescence was determined by analysis using a FACSCanto II™ instrument (BD Biosciences). To assess the effect of mAbs or BsAbs on ligand binding, CD27 expressing Ramos cells (ATCC) or HEK293-PD-L1 cells were briefly pre-incubated with the mAbs, BsAbs or controls, followed by the addition of 0.5 µg/ml human CD70-biotin (US Biological) or 0.5 µg/ml PD-1-biotin (R&D Systems), respectively. Binding of biotinylated ligands was detected with streptavidin PE (SA–PE) and analyzed on a FACSCanto II™ instrument.

### CD27 agonist assays

#### NFκB reporter assay

A stable cell line was developed from HEK293 NFκB-luciferase reporter cell line (Signosis, Inc.) transfected with human CD27. Cells were incubated with mAbs or BsAb for 6 h at 37 °C, 6% CO_2_. Luciferase was detected with the Luciferase Assay System (Promega).

#### Human T cell costimulation

Ninety-six well tissue culture plates were prepared by adding 1 µg/ml anti-CD3 mAb (OKT3- eBioscience), and/or 2 µg/ml recombinant human PD-L1 and coated overnight at 4 °C. After washing the wells with PBS, 100,000 CD3^+^ cells isolated by magnetic bead separation from peripheral blood mononuclear cells (PBMC) were added to each well in media. The plates were incubated for 3 days at 37 °C and 5% CO_2_, and supernatants were harvested and analyzed for IL-2 or IFN-γ production by ELISA (R&D Systems).

### Cell-based PD-1 signaling assay

The effect of the BsAb on blockade of PD-1 signaling was performed per manufacturer’s instructions with a cell-based method in which blocking PD-1 signaling allows T cell receptor (TCR) activation and induces luminescence via the NFAT pathway (Promega). Luminescence was detected by the addition of Bio-Glo reagent and quantitated on a PerkinElmer Victor X luminometer.

### Mixed lymphocyte reaction (MLR)

Human PBMCs were isolated from buffy coats using Ficoll separation, and CD4^+^ cells were further isolated using magnetic bead separation (Miltenyi). Monocyte-derived dendritic cells (DC) were generated from PMBCs by adhering to plastic and then cultured for 7 days in RPMI medium containing 10% FBS, 10 ng/ml IL-4 plus 100 ng/ml GM-CSF (R&D Systems). Cells were harvested and confirmed to be 80% DCs by expression of CD11c. The CD4^+^ cells and DCs from allogeneic donors were co-incubated at a 10:1 ratio in the presence of mAb or BsAb for 3 days. Supernatants were harvested and analyzed for IL-2 or IFN-γ production by ELISA (R&D Systems).

### Antibody-dependent cellular cytotoxicity (ADCC)

ADCC activity was evaluated using a commercially available ADCC Reporter Bioassay Kit (Promega). This assay indirectly measures ADCC through quantitation of the FcγRIIIa receptor activity. Target cells included tumor cell lines Ramos (CD27^+^) and MDA-MB-231 (PD-L1^+^) (ATCC) or HEK293 cell lines expressing either CD27 or PD-L1.

### Enhancement of vaccine-specific T cell responses

The huCD27-Tg mice [19] received intraperitoneal (i.p.) administrations of 0.05 mg of BsAb or mAbs and 5 mg of ovalbumin (OVA) (Sigma-Aldrich). After 1 week, spleen cells were harvested and ELISPOT analysis was performed with and without 2 µg/ml SIINFEKL peptide (GenScript) incubated overnight in IFN-γ Ab-coated 96-well filtration plates (Sigma-Aldrich). Spots were developed by using an IFN-γ antibody set and a 3-amino-9-ethylcarbazole substrate (BD Biosciences) and counted by ZellNet Consulting, Inc.

### Mouse tumor models

For the syngeneic lymphoma model, huCD27-Tg Balb/C mice were injected intravenously (i.v.) with BCL1 cells (1 × 10^6^) on day 0, followed by i.p. injection of mAbs or BsAbs (0.2 mg) on days 5 or 7. Mice were observed daily for survival. Xenograft tumor studies were performed using the human B cell lymphoma cell line Raji (ATCC) with 0.5 × 10^6^ cells implanted subcutaneously (s.c.) on day 0 into the flanks of SCID mice cells followed by i.p. administration of mAbs or BsAbs (0.1 mg) on days 5, 8, 12, 15, 19, and 22. Tumors were measured by caliper twice per week, and mice were euthanized according to pre-defined endpoint criteria.

### Pilot non-human primate study

Three male cynomolgus monkeys (Citoxlab, Stilwell KS) received a single 7 mg/kg slow bolus i.v. injection (2–3 min) of CDX-527, via a cephalic catheter. Animals were followed for 21 days. Evaluations included clinical signs, body temperature, clinical pathology parameters (hematology, coagulation, clinical chemistry and urinalysis), and toxicokinetic parameters. Body weights were recorded once prior to BsAb administration and weekly thereafter. This was designed as a survival study with no planned necropsy.

Quantitation of CDX-527 concentration and anti-drug antibodies (ADA) was performed using a Mesoscale Discovery platform (MSD). For pharmacokinetics (PK), the plates were coated with a human CD27-Fc. The bound CDX-527 was detected by adding human PD-L1-msFc and a ruthenium-labeled SULFO-Tag F(ab’)_2_ (Mesoscale diagnostics) goat anti-mouse IgG (Fc-specific) followed by tripropylamine. The ADA assay used streptavidin-coated plates to capture biotinylated CDX-527. Serum samples were then added, and reactive antibodies were detected with ruthenium-conjugated CDX-527 and tripropylamine.

### Statistical analysis

Statistical significance was evaluated using two-way ANOVA or paired Student’s t-test as appropriate. For tumor survival studies, the Mantel-Cox test was used.

## Results

### Characterization of novel CD27 and PD-L1 antibodies

**Fig. 1:**
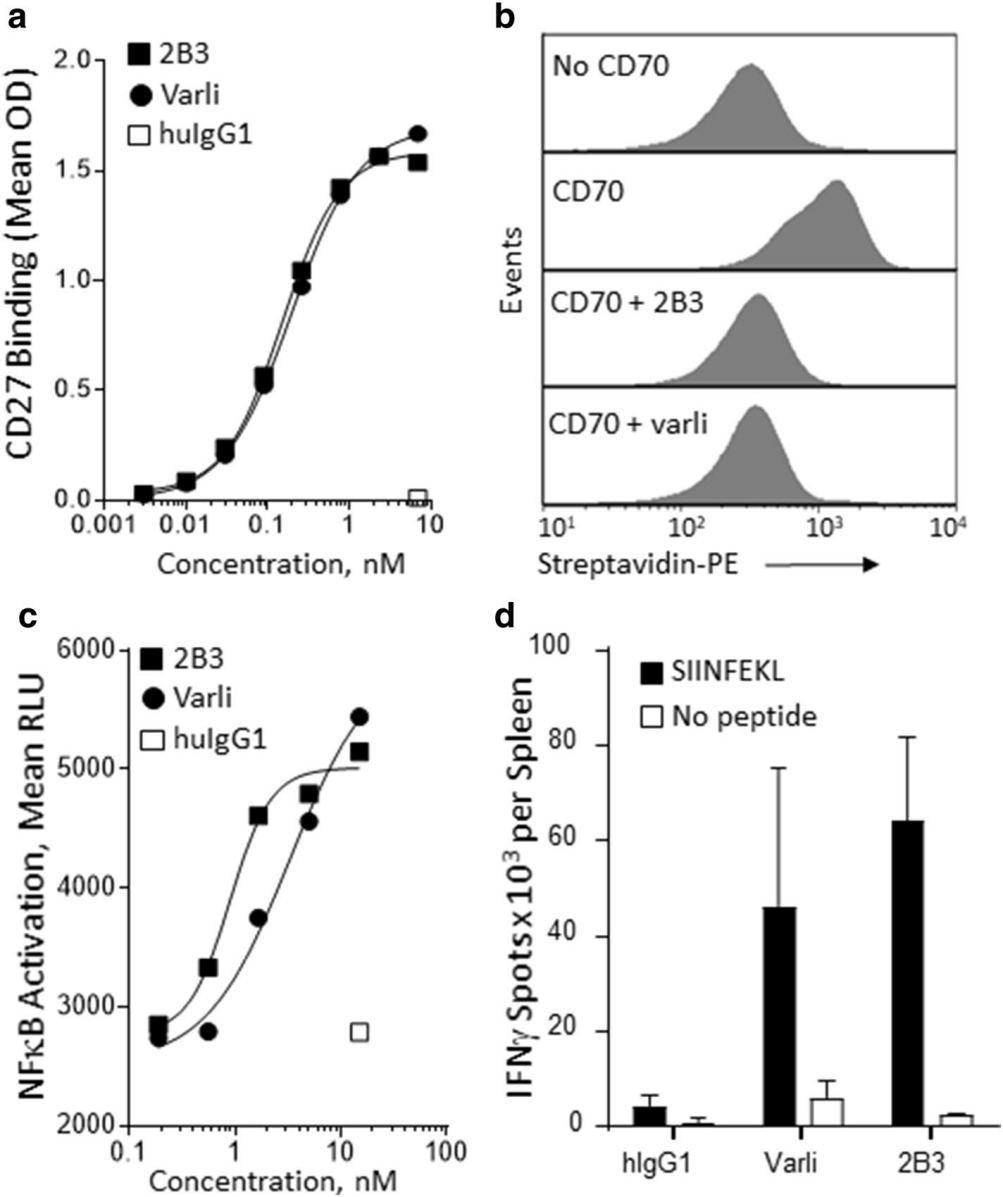
Characterization of anti-CD27 mAb 2B3**. a** ELISA binding to human CD27. Binding detected with an HRP-labeled goat anti-human IgG (Fc-specific) antibody. **b** 2B3 blocks CD70 binding to Ramos cells expressing CD27. Ramos cells were incubated with mAbs followed by the addition of CD70-biotin. CD70-biotin was detected with streptavidin-PE. **c** CD27 agonist activity of 2B3. NFκB reporter 293 cells expressing CD27 were incubated with mAbs. Luciferase production was detected with Luciferase Assay System (Promega) **d** 2B3 enhances antigen-specific T cell responses to OVA immunization in vivo. huCD27-Tg mice (*n* = 3 per group) were injected i.p. with 5 mg of OVA together with 50 µg mAbs and spleens were collected 7 days later. ELISPOT assays were performed in triplicate from animals treated with hIgG1, varlilumab (Varli), or 2B3, and cultured in the presence (black bars) or absence (white bars) of SIINFEKL peptide. Shown are the mean number of spot-forming units per spleen (± SD)

Antibodies to CD27 and PD-L1 were generated as described in materials and methods. The CD27 mAb designated 2B3 was selected for development of the BsAb based on its functional characteristics. As shown in Fig. 1a–d, 2B3 mAb and varlilumab showed a comparable concentration dependence of antigen binding, CD27 agonist activity using an NFκB reporter cell line, and ability to enhance antigen-specific T cell responses in huCD27-Tg mice. Similar to varlilumab, the 2B3 mAb blocks binding of the CD70 ligand to CD27.

**Fig. 2:**
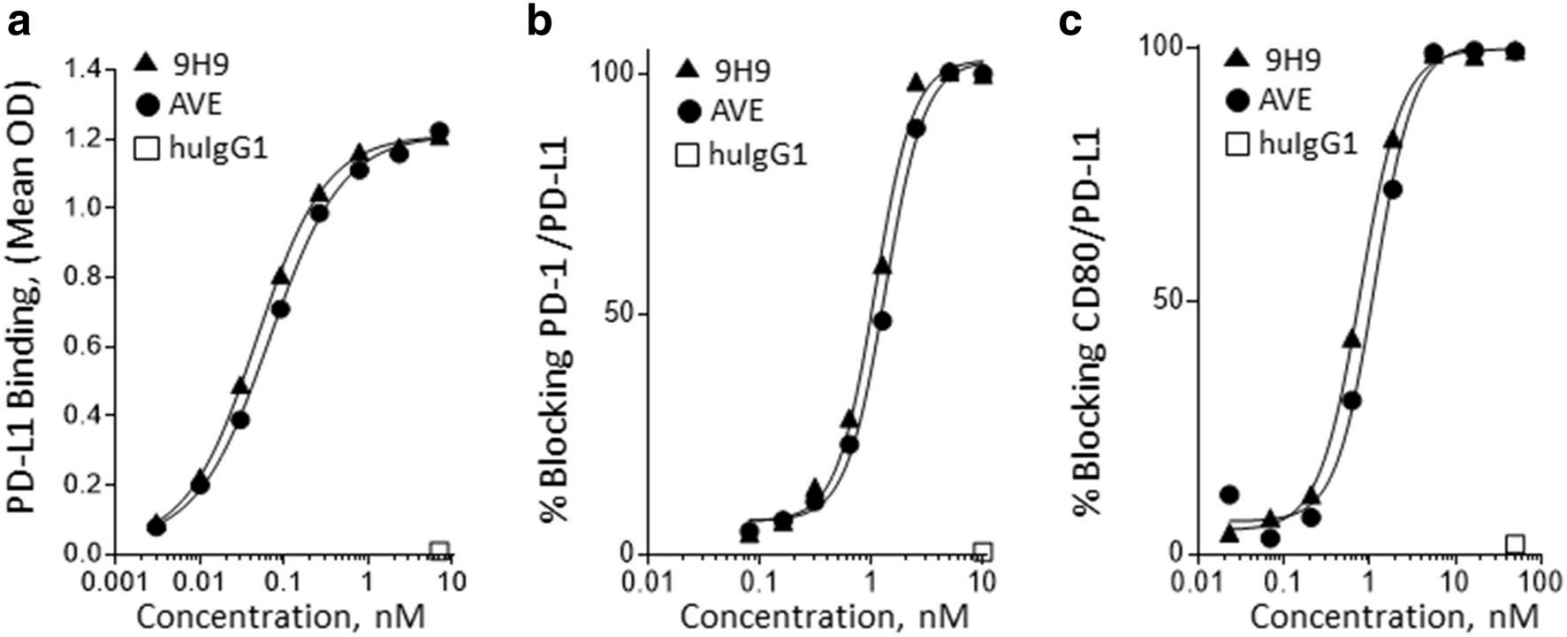
Characterization of anti-PD-L1 mAb 9H9**. a** ELISA binding to human PD-L1 by 9H9 and avelumab (AVE). Binding was detected with an HRP-labeled goat anti-human IgG (Fc-specific) antibody. **b** 9H9 blocks PD-1 binding to PD-L1. HEK293-PD-L1 cells were incubated with samples and PD-1-biotin. Binding detected with a streptavidin–phycoerythrin (SA–PE) conjugate. **c** 9H9 blocks CD80 binding to PD-L1. Sample and PD-L1-biotin were incubated on a CD80-coated plate. Binding was detected with a streptavidin–horseradish peroxidase (SA–HRP) conjugate

The 9H9 PD-L1 mAb was also selected for use in the BsAb based on its functional characteristics. For comparison, the anti-PD-L1 mAb, avelumab, was cloned and expressed by introducing the V_H_ and V_L_ coding regions into the same human IgG1κ backbone used for mAb 9H9. The 9H9 mAb and avelumab demonstrated a similar concentration dependence in binding to PD-L1, inhibition of PD-1 binding to PD-L1, and inhibition of CD80 binding to PD-L1 (Fig. 2a–c).

### Development of the CDX-527 BsAb

**Fig. 3:**
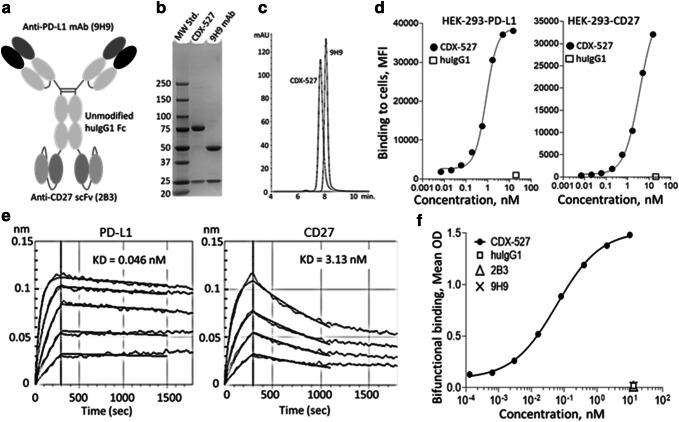
Analytical characterization of CDX-527**. a** Illustration of the CDX-527 design format. **b** SDS-PAGE of molecular weight standards (lane 1), CDX-527 (lane 2) and 9H9 mAb (lane 3) run under reducing conditions. **c** Overlay of HPLC profiles of CDX-527 and 9H9 mAb on a TSK3000 SEC column showing relative size homogeneities and molecular weights. **d** Binding to HEK293 cells expressing PD-L1 or CD27 as measured by flow cytometry. **e** Binding affinities of CDX-527 to individual target antigens. Sensograms of bio-layer interferometry analysis using anti-human IgG-Fc sensors to capture CDX-527 followed by increasing concentrations of soluble PD-L1 or CD27, with respective KD values. **f** Bifunctional ELISA measures simultaneous binding of both CD27 and PD-L1 to CDX-527. Wells coated with huCD27 and blocked, followed by mAbs or BsAb and then a soluble PD-L1 fused to mouse Fc protein. Detection employed a goat anti-mouse IgG (Fc-specific)-HRP

We selected a whole IgG1-scFv format (Fig. 3a) for the CDX-527 BsAb construct with bivalent binding to both PD-L1 and CD27 to maintain high affinity binding, and with unmodified IgG1 constant domains to allow Fcγ receptor (FcγR) and neonatal Fc receptor (FcRn) interactions to promote cross-linking of CD27 and an IgG1-like circulation half-life, respectively. To build off a PD-L1 mAb backbone, the expression construct encoded the full length 9H9 IgG1 heavy and light chains and the scFv of the 2B3 mAb genetically linked in *V*_L_–*V*_H_ orientation to the C-terminus of the 9H9 mAb heavy chain. We also made the bispecific construct with the 2B3 scFv in the *V*_H_–*V*_L_ orientation, but it exhibited degradation and lower activity in the bifunctional ELISA compared to *V*_L_–*V*_H_ (not shown) and was therefore not pursued.

Analytical characterization was performed on CDX-527 purified from CHO cells stably transfected with the expression plasmid. Reducing SDS-PAGE of CDX-527 revealed the anticipated greater molecular weight of the heavy chain (approximately 75 kDa) relative to 9H9 due to the scFv fragment (Fig. 3b). Size exclusion chromatography (SEC) by HPLC demonstrated the BsAb product to be > 90% monomer with an estimated size of approximately 200 kDa for CDX-527 (Fig. 3c), and the BsAb demonstrated good manufacturing characteristics including no significant changes observed in accelerated stability studies at 40 °C (not shown). The BsAb retained high affinity binding to HEK293 cells transfected with either PD-L1 or CD27 by flow cytometry and to recombinant human PD-L1 and CD27 as determined by bio-layer interferometry (Fig. 3d–e) and also bound to human Fcγ receptors and the neonatal FcRn under low pH conditions (Supplementary Fig. 1). Simultaneous binding to CD27 and PD-L1 by CDX-527 was demonstrated using a bifunctional ELISA (Fig. 3f). Similar bifunctional binding curves were observed when the ELISA was performed in the reverse orientation and the binding to cells expressing the antigens was confirmed by flow cytometry (data not shown).

### CDX-527 activates T cells more potently than its parental antibodies

**Fig. 4:**
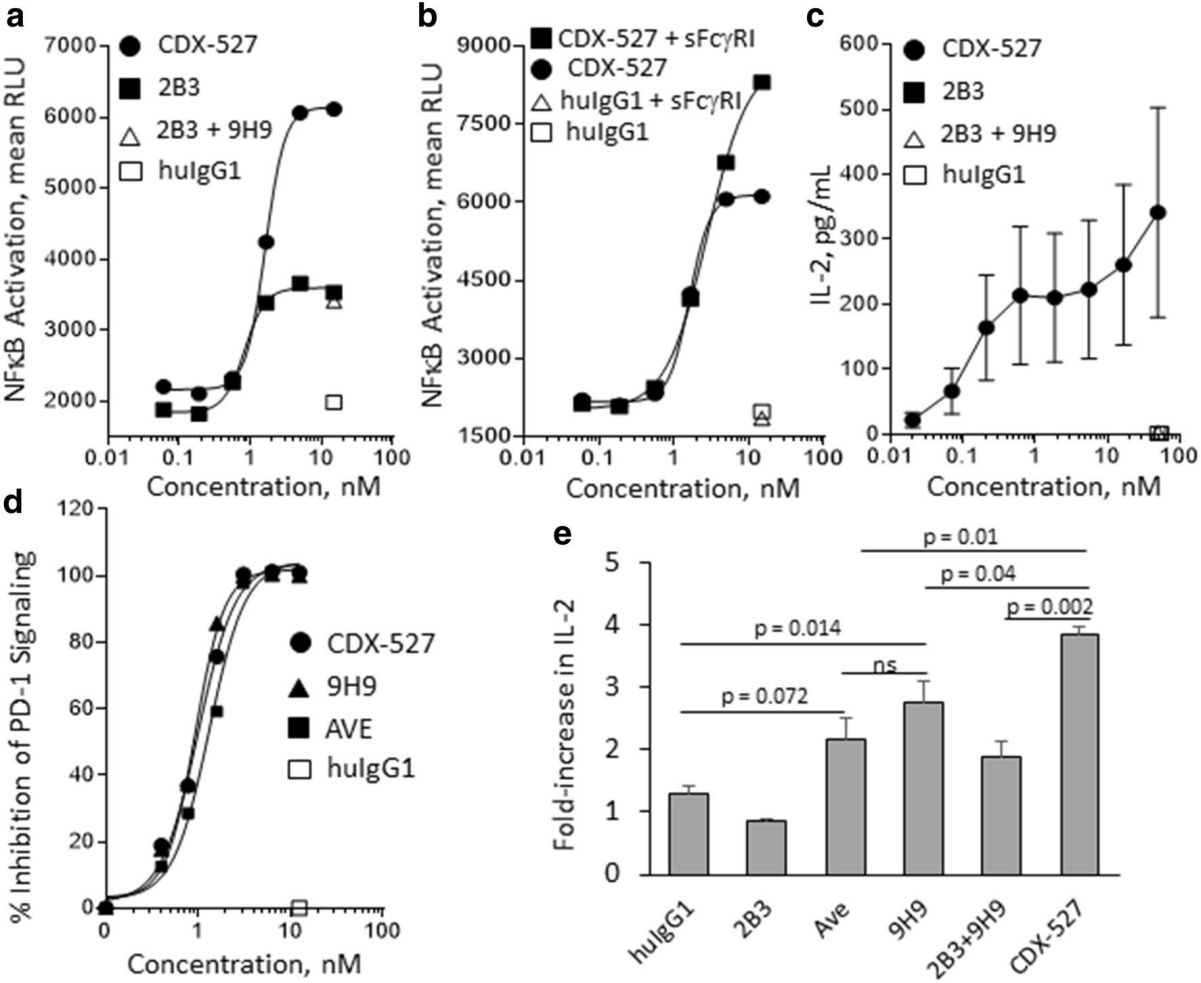
In vitro functional activities of CDX-527 compared to parental mAbs**.** CD27 agonist activity is enhanced by cross-linking through **a** PD-L1 and **b** FcγR1. CD27-NFκB reporter HEK293 cells were incubated with mAbs or BsAb either alone or in the presence of human soluble FcγR1 (sFcγR1) as indicated. Luciferase production was detected with the Luciferase Assay System (Promega). **c** T cell activation requires TCR signaling and cross-linking. Primary human T cells from 5 donors with mAbs or BsAb as indicated were added to a plate previously coated with suboptimal OKT3 and soluble PD-L1. Supernatant was harvested at 72 h and analyzed for IL-2 concentration by ELISA and results are shown as mean values (± SEM, *n* = 5). **d** PD-1 signal blockade was measured with a commercially available cell-based assay from Promega. The PD-1 effector cells and PD-L1 cells were co-cultured in the presence of mAbs or BsAb followed by detection of luminescence. **e** CDX-527 enhances T cell activation in MLR assays. CD4^+^ T cells from 3 donors and allogeneic DCs were co-cultured (MLR) in the presence of mAbs or BsAb for 3 days. Supernatant was harvested and analyzed for IL-2 by ELISA and is shown as the mean fold-increase in IL-2 concentration (± SEM, *n* = 3)

CDX-527 induced greater agonist activity than the parental 2B3 mAb using the CD27-NFκB reporter cell line (Fig. 4a). The enhanced activity is likely due to the expression of PD-L1 on the reporter cells that would promote cross-linking and more potent signaling (Supplementary Fig. 2). The reporter cells do not express Fc receptors; however, the CD27 agonist activity of CDX-527 could be further augmented with the addition of recombinant soluble FcγRI (sFcγRI, Fig. 4b) which contains higher-order multimers as observed by HPLC (data not shown).

We next looked at the ability of the BsAb to costimulate primary human T cells. In these experiments, microtiter wells were coated with suboptimal amounts of OKT3 mAb for TCR stimulation and PD-L1 protein to capture CDX-527 and promote cross-linking and CD27 signaling. CDX-527 induced concentration-dependent T cell activation as shown by IL-2 production (Fig. 4c). T cell activation was also demonstrated by IFN-γ production and proliferation (data not shown). Under these conditions, the 2B3 mAb, or the combination of 2B3 and 9H9 mAbs, did not result in T cell activation. Further, the BsAb only induced T cell activation if both OKT3 mAb and PD-L1 protein were coated to the plate (Supplementary Fig. 3), confirming that TCR stimulation and cross-linking are both required for CDX-527-mediated T cell costimulation.

Inhibition of PD-1 signaling by CDX-527 was demonstrated in a cell-based reporter assay in which CDX-527 and the 9H9 mAb induced reporter activation with IC_50_ values of 1.00 nM and 0.93 nM, respectively (Fig. 4d). Finally, in T cell activation assays promoted by co-culture with allogeneic DCs (MLR), CDX-527 induced IL-2 more potently than PD-L1 blockade with either avelumab, the 9H9 mAb, or the combination of 9H9 and 2B3 mAbs (Fig. 4e). Despite the expression of Fc receptors on DCs, 2B3 mAb was unable to provide T cell costimulation either alone or in combination with 9H9 mAb.

### Effector function of CDX-527

**Fig. 5:**
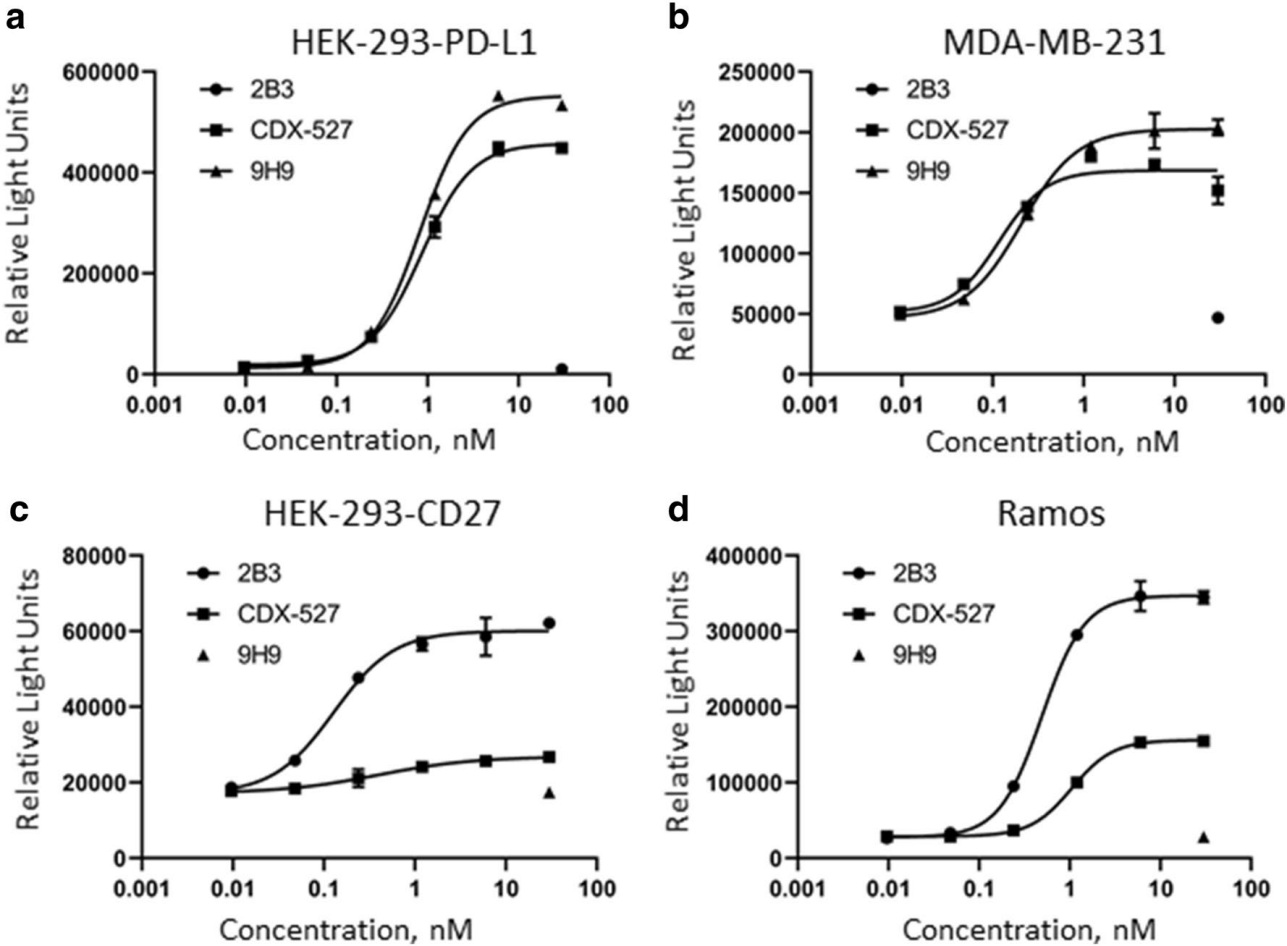
CDX-527 mediated ADCC. Indicated cell lines expressing PD-L1 (**a**, **b)** or CD27 (**c**, **d)** were mixed with effector reporter cells in the presence of increasing concentrations of CDX-527, 9H9 or 2B3 for 6 h at 37 °C. ADCC activity was measured through NFAT-driven luciferase expression in effector cells. Mean (± SD) relative light units for duplicates in a single experiment are shown. The experiment was repeated with similar results

While the BsAb was designed to enhance T cell immunity, as described above, CDX-527 binds to activating FcγRs and thereby can potentially mediate effector function such as ADCC against certain tumor cells overexpressing PD-L1 or CD27. CDX-527 induced ADCC in tumor cell lines endogenously expressing PD-L1 or CD27, or cells transfected with the receptors, though the BsAb was somewhat less potent than the parental 2B3 mAb with the CD27 expressing cells (Fig. 5a–d). As a confirmation of target specificity, 9H9 induced ADCC only in PD-L1 but not in CD27 expressing cells, while 2B3 induced ADCC in CD27 but not PD-L1 expressing cells. CDX-527 did not demonstrate measurable complement mediated cytotoxicity against the same cell lines (data not shown).

### Enhancement of vaccine response in vivo with a surrogate BsAb construct

**Fig. 6:**
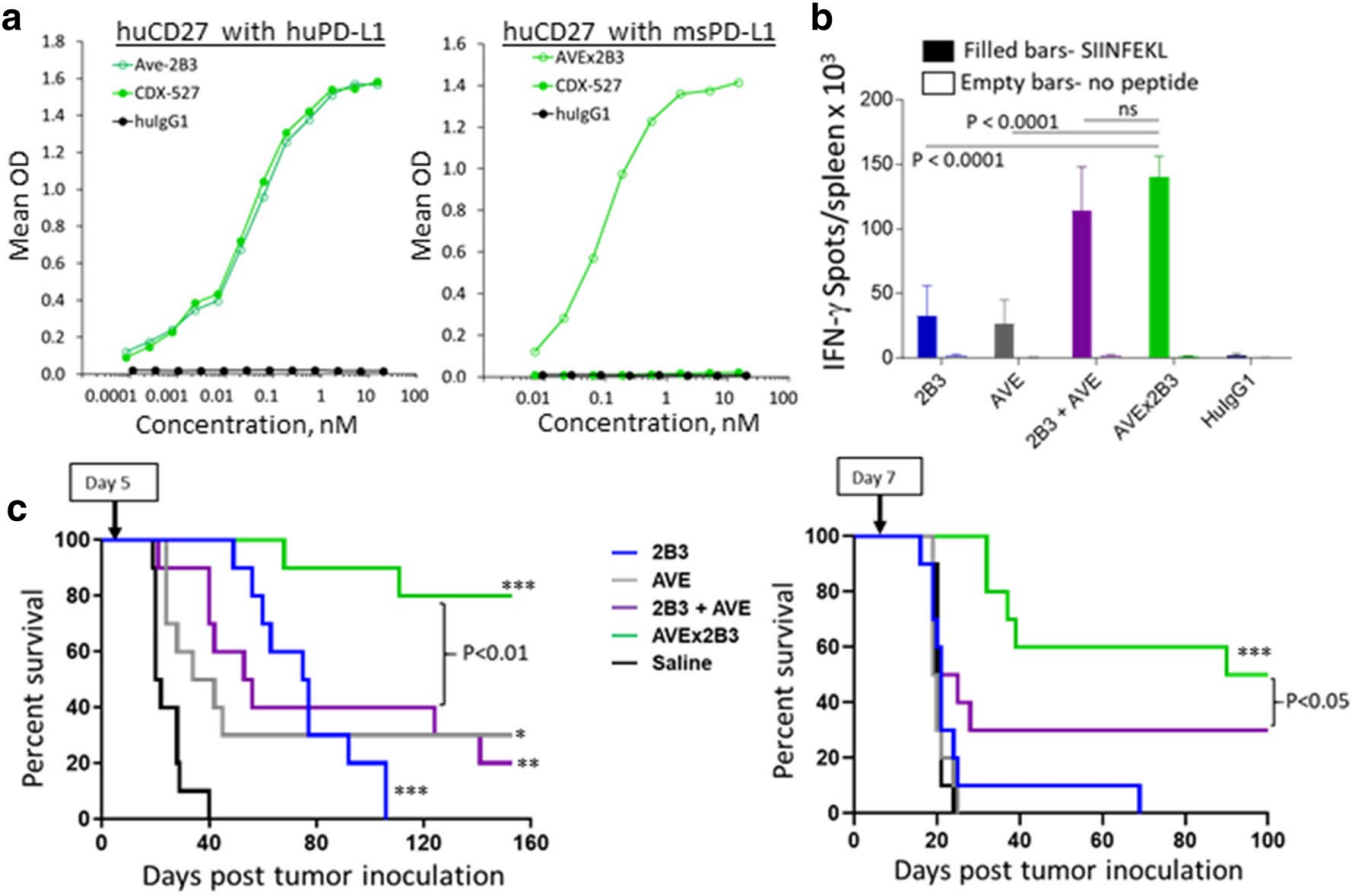
Enhanced vaccine response and antitumor activity of BsAb. **a** CDX-527 binds human CD27 and human PD-L1 whereas AVEx2B3 binds human CD27 and both human and mouse PD-L1. Bifunctional ELISA assays used wells coated with human CD27 and probed with recombinant human or mouse PD-L1 proteins fused to mouse Fc. Binding was detected with an HRP-labeled goat anti-mouse IgG (Fc-specific) antibody. **b** AVEx2B3 enhances antigen-specific T cell responses to OVA immunization in vivo. ELISPOT assays were performed in triplicate using huCD27-Tg mice (*n* = 5 per group) immunized with OVA as described in Fig. 1d and cultured in the presence or absence of SIINFEKL peptide overnight. Shown are the mean (± SD) number of IFN-γ-specific spot forming units per spleen. **c** AVEx2B3 prolongs survival in a syngeneic BCL1 lymphoma mouse model. Groups of 10 huCD27-Tg mice were inoculated i.v. with 1 × 10^6^ BCL1 cells on day 0. A single treatment of 0.2 mg of mAb or BsAb was given i.p. either on day 5 (left graph) or day 7 (right graph). The p value for the BsAb versus the combination of mAbs is shown. The asterisks indicate treatments that are statistically significant relative to control, * *p* < 0.01, ** *p* < 0.001, *** *p* < 0.0001

We have previously described mice that were engineered to express human CD27 (huCD27-Tg mice), which allow in vivo use of the 2B3 mAb [19]. The 9H9 mAb binds human and macaque PD-L1 but does not cross-react with rodent PD-L1 (Supplementary Fig. 4). Therefore, we developed a surrogate BsAb with a mouse cross-reactive PD-L1 mAb to evaluate in huCD27-Tg mice. The V_H_ and V_L_ sequences of 9H9 in CDX-527 were replaced with those from the anti-PD-L1 mAb, avelumab, which binds equally well to human and mouse PD-L1 [27]. The surrogate BsAb, designated AVEx2B3, bound equally well as CDX-527 in the bifunctional ELISA with human CD27 and PD-L1 proteins, and retained similar binding to mouse PD-L1 (Fig. 6a).

The ability of AVEx2B3 to enhance antigen-specific T cell responses was tested by administering OVA protein to huCD27-Tg mice together with the BsAb or controls. The expansion of OVA-specific T cells was determined by IFN-γ ELISPOT in response to OVA peptide (SIINFEKL) stimulation of spleen cells harvested 1 week after vaccination (Fig. 6b). Both the anti-CD27 mAb and anti-PD-L1 mAb increased the expansion of OVA-specific CD8 T cells compared to isotype control. However, the combination of the mAbs and the surrogate BsAb significantly enhanced the OVA-directed cytotoxic T lymphocyte (CTL) response as compared to the single mAb treatment.

### Enhancement of antitumor activity in vivo with AVEx2B3

We next tested AVEx2B3 in the disseminated BCL1 lymphoma model. When animals were administered a single dose of AVEx2B3 or control antibodies (0.2 mg/mouse) on day 5 after tumor inoculation, we observed improved survival in all treatment groups, but only the BsAb led to long-term survival in the majority of animals (Fig. 6c). Delaying treatment until 1-week post-tumor inoculation allowing more tumor growth and thereby increasing resistance to treatment, abrogated the benefit in the monotherapy groups, yet AVEx2B3 remained similarly effective (Fig. 6d).

### Direct anti-lymphoma activity

**Fig. 7:**
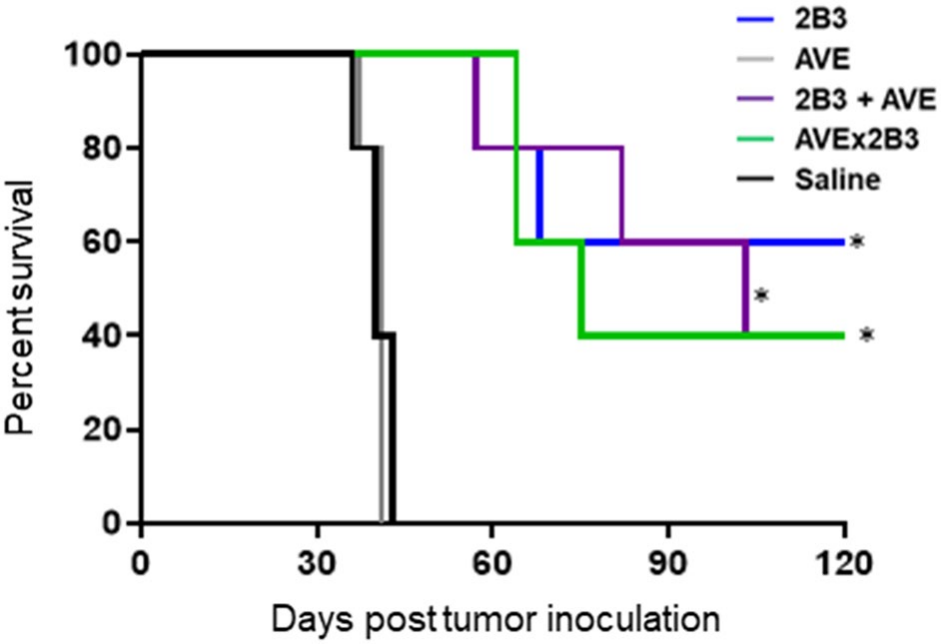
Direct killing of a CD27-expressing lymphoma in an immunodeficient mouse model. Groups of SCID mice (*n* = 5 per group) were inoculated s.c. with 0.5 × 10^6^ Raji cells on day 0. The mAbs or BsAb were dosed at 0.1 mg by i.p. injection on days 5, 8, 12, 15, 19 and 22. Treatment with 2B3 monotherapy, in combination with AVE, and the bispecific AVEx2B3 all extended survival compared to AVE or saline control (*p < 0.01). There is no statistical difference between the 2B3, 2B3 + AVE and AVEx2B3 groups. Representative of 2 separate experiments

While the BsAb targets CD27 for costimulation of T cells to enhance general antitumor activity, an alternative mechanism involving ADCC enabled by FcR binding may also contribute to antitumor effects in certain lymphoid tumors expressing CD27. Therefore, we investigated the antitumor activity of the surrogate BsAb in SCID mice transplanted with Raji tumor cells that express high levels of CD27. As shown in Fig. 7, the 2B3 mAb, the combination of 2B3 and AVE mAbs, and the AVEx2B3 BsAb each had similar antitumor activity. There was no additional benefit from including the PD-L1 mAb in the combination or in the BsAb.

### Pilot non-human primate study

**Fig. 8:**
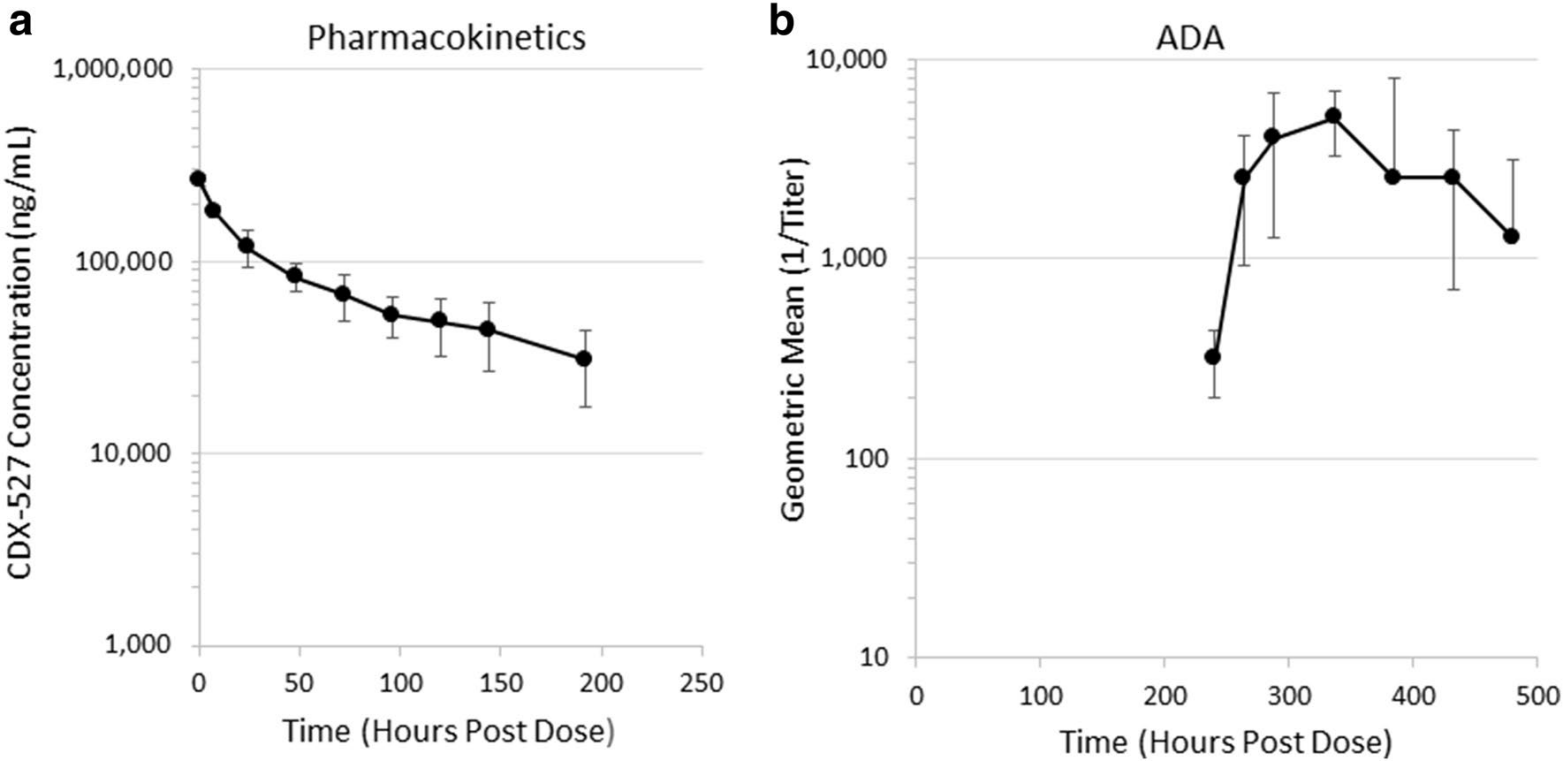
Pharmacokinetics and immunogenicity of CDX-527 in cynomolgus macaques. Serum levels of CDX-527 and ADA were determined using an MSD platform as described under Materials and Methods. **a** CDX-527 concentration in serum of cynomolgus macaques over time after a bolus i.v. injection of 7 mg/kg CDX-527. **b** The corresponding ADA response. Values represent mean (**a**) or geometric mean (**b**) ± SD, *n* = 3

CDX-527 was administered to cynomolgus macaques to have a preliminary assessment of the PK and in-life tolerability-related endpoints. Three male cynomolgus monkeys received a single 7 mg/kg slow bolus i.v. injection of CDX-527 and were followed for 21 days. There were no CDX-527-related clinical observations including body weights and body temperature. Clinical chemistry and hematological parameters remained unchanged or were considered non-adverse. Pharmacokinetic (PK) analysis was performed on samples only up to day 8, as each animal rapidly developed anti-CDX-527 antibodies at subsequent time points, which impacted clearance and accurate PK assessment (Fig. 8). From this small study, the approximate mean t_1/2_ value for terminal disposition of CDX-527 was calculated to be 5.31 days.

## Discussion

We developed a BsAb, designated CDX-527, based on the strong scientific rationale for coupling CD27 costimulation with PD-1 blockade, which included preclinical data demonstrating complementary and synergistic effects in tumor models [22] and clinical data supporting safety and efficacy at the same dose and regimen for each antibody [24, 25]. To generate the BsAb, we initially developed and characterized novel fully human antibodies to CD27 (mAb 2B3) and PD-L1 (mAb 9H9) that were primarily selected for CD27 agonist activity and inhibition of PD-1 signaling, respectively. The 9H9 antibody was also selected for its potent inhibition of CD80 binding to PD-L1, which may reduce the recently revealed cis-interactions between CD80 and PD-L1 on antigen presenting cells (APC) thereby resulting in more available CD80 for costimulation through CD28 [28]. CDX-527 was designed as a tetravalent IgG1-scFv construct that is produced and purified by standard technologies applied for mAbs. As expected, CDX-527 retained the high-affinity binding to PD-L1 and to CD27, similar to the parental mAbs, and could engage each target simultaneously as demonstrated using a bifunctional ELISA.

Efficient antibody-mediated CD27 signaling requires receptor clustering optimally achieved by crosslinking the CD27 mAb, e.g., via FcγR binding [29, 30]. Using a CD27-signaling reporter cell line that expresses low levels of PD-L1 and no FcR, we demonstrated that CDX-527 indeed was a more potent agonist than the parental CD27 mAb 2B3. The CD27 agonist activity of CDX-527 was further enhanced through FcR cross-linking, demonstrating that both PD-L1 and Fc receptor cross-linking can contribute to CD27 agonism with the BsAb.

The enhanced CD27 agonist activity of CDX-527 relative to mAb targeting was further demonstrated using in vitro T cell activation assays measured primarily by IL-2 production. Direct CD27 costimulation with the BsAb was achieved when human T cells were cultured on plates precoated with the anti-CD3 mAb OKT3 and PD-L1, but not achieved with the parental CD27 or PD-L1 mAbs even when combined. CDX-527 is efficiently cross-linked through binding to PD-L1, whereas the parental CD27 mAb is not cross-linked, and PD-1 blockade using the parental PD-L1 mAb, in the absence of T cell activation, does not have a measurable effect. As observed previously for CD27 costimulation [20, 29], CDX-527 required both TCR stimulation and cross-linking to result in costimulation of T cells.

In MLR assays, addition of CDX-527 led to significantly greater T cell activation than the individual or combined CD27 and PD-L1 mAbs. Despite the presence of FcR on the APCs, the CD27 mAb did not enhance T cell activation either alone or in combination with the PD-L1 mAb. Presumably, in this setting the FcR-mediated cross-linking of the 2B3 mAb is suboptimal for good agonist activity. In contrast, PD-1 blockade with the PD-L1 mAb did enhance T cell activation in the MLR studies, as has been reported with other PD-(L)1 inhibitors [31–33]. The greater activity of CDX-527 relative to the PD-L1 mAbs is most likely due to more efficient cross-linking of the BsAb that can be mediated through both FcR and PD-L1 interactions.

To study in vivo activity of the BsAb, we leveraged huCD27 transgenic mice [19] and the murine cross-reactivity of the PD-L1 mAb, avelumab. The surrogate BsAb, AVEx2B3, demonstrated potent activity in vivo. As previously reported, CD27 agonist mAbs significantly boost the number of antigen-specific CD8 effector cells in an FcR-dependent manner when combined with a vaccine [19] and the response is further increased in the context of PD-1/PD-L1 blockade [22]. AVEx2B3 was more effective in boosting the CD8 effector T cell response to vaccine than either the CD27 or PD-L1 mAb, although its activity did not exceed the strong effect seen with the combination of the parental mAbs at the doses tested.

The superiority of the BsAb over the combination of CD27 and PD-L1 mAbs was clearly evident in a disseminated BCL1 lymphoma model. BCL1 expresses PD-L1 but not CD27 which would allow costimulation of T cells in the huCD27-Tg mice, enhancement of CD27 costimulation by crosslinking through PD-L1 in addition to FcR, and direct tumor killing through PD-L1. Other syngeneic tumor models in huCD27-Tg mice have shown that reductions in CD27-expressing Treg cells using varlilumab can also contribute to anti-tumor activity [21]. We have previously shown that the efficacy of anti-CD27 therapy in the BCL1 model correlated with the level of CD27 agonism more strongly than Treg reductions [21], suggesting that the additional cross-linking through PD-L1 enhanced CD27 signaling and antitumor activity of the BsAb.

In addition to the immune modulating activities of CD27 agonism and PD-1 blockade, the BsAb may have direct antitumor activity due to its capacity to bind FcR. We observed similarly potent ADCC with CDX-527 and the 9H9 mAb of PD-L1 expressing tumor cells, a mechanism that has been associated with antitumor activity for PD-L1 mAbs that can engage activating FcγRs [34, 35]. Although the in vitro ADCC activity of CDX-527 was less potent than the 2B3 mAb, AVEx2B3 and 2B3 both similarly improved the survival of immunodeficient mice transplanted with the human Burkitt’s lymphoma line Raji that has high CD27 expression. The direct antitumor activity against Raji cells likely involves innate ADCC effector cells such as NK or macrophages [36] but is unlikely to involve complement meditated cytotoxicity based on our in vitro data.

The inclusion of the intact human IgG1 Fc domain in the BsAb design was important for achieving mAb-like PK through FcRn binding. This was demonstrated in a small study performed in macaques that estimated the elimination half-life (t_1/2_) for CDX-527 to be 5.31 days. In these animals, no obvious toxicity was observed, although the strong ADA response makes longer-term evaluations challenging in this species. Immunogenicity in non-human primates, however, is not considered predictive of immunogenicity in humans [37]. Collectively, these data support CDX-527 as a promising approach to enhance the activity of PD-1 blockade, and activities to support evaluation of CDX-527 in cancer patients are underway. In addition to expanding the clinical benefits of PD-1 blockade, future combinations of CDX-527 with in situ vaccination strategies that induce immunogenic cell death [38], including certain chemotherapies [39] and radiation treatment [40, 41], may further improve outcomes.

### Electronic supplementary material

Below is the link to the electronic supplementary material.

Supplementary file1 (PDF 244 kb)

## Data Availability

All data generated or analyzed during this study are included in this published article and its supplementary information files.

## Abbreviations

ADA: Anti-drug antibody
ADCC: Antibody-dependent cell-mediated cytotoxicity
APC: Antigen presenting cell
AVE: Avelumab
BsAb: Bispecific antibody
CHO: Chinese hamster ovary
CTL: Cytotoxic T lymphocyte
DC: Dendritic cells
EBV: Epstein–Barr virus
ELISA: Enzyme-linked immunosorbent assay
FBS: Fetal bovine serum
FcR: Fc receptor
FcγR: Fcγ receptor
FcRn: Neonatal Fc receptor
HPLC: High-pressure liquid chromatography
HRP: Horseradish peroxidase
huCD27-Tg: Human CD27-transgenic
i.p.: Intraperitoneal
i.v.: Intravenous
mAb: Monoclonal antibody
MLR: Mixed lymphocyte reaction
MSD: Mesoscale Discovery
MSI: Microsatellite instability
MTD: Maximum tolerated dose
OVA: Ovalbumin
PBMC: Peripheral blood mononuclear cell
PBS: Phosphate-buffered saline
PE: Phycoerythrin
PK: Pharmacokinetics
SA: Streptavidin
scFv: Single-chain variable fragment
SCID: Severe combined immune-deficient
s.c.: Subcutaneous
SEC: Size exclusion chromatography
TCR: T cell receptor
TNFR: Tumor necrosis factor receptor
Treg: Regulatory T cells
