# Supplementary material for: Development of CDX-527: a bispecific antibody combining PD-1 blockade and CD27 costimulation for cancer immunotherapy
Source: Cancer Immunol Immunother. 2020 May 25;69(10):2125–37. doi: 10.1007/s00262-020-02610-y (PMC7511290; doi:10.1007/s00262-020-02610-y)
Supplement: Supplementary file 1 — Supplementary file1 (PDF 244 kb) [file 262_2020_2610_MOESM1_ESM.pdf]

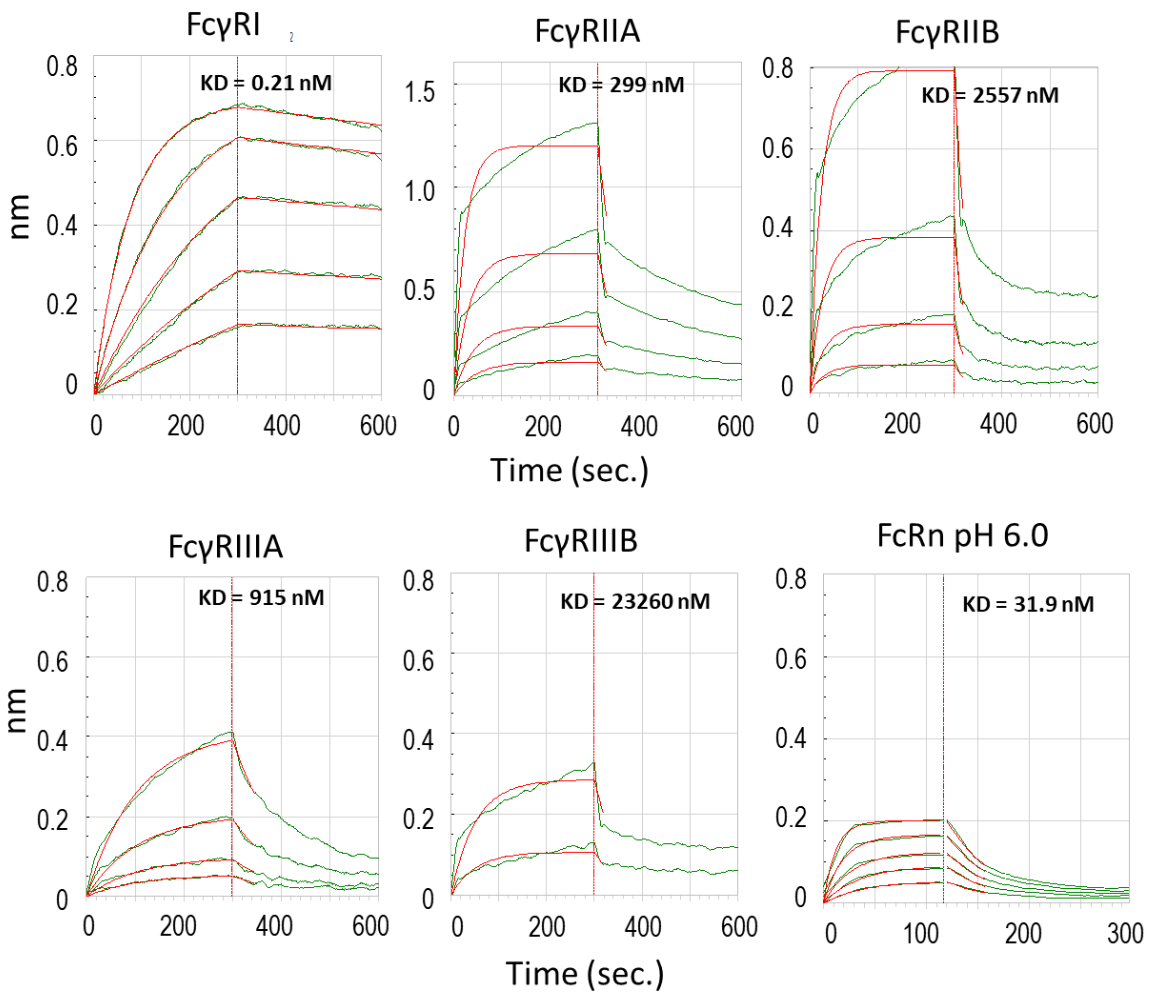

Supplementary Fig. 1. CDX-527 binding to human Fc receptors. Sensorgrams of bio-layer interferometry analysis using streptavidin sensors to capture respective biotinylated soluble human Fc receptors followed by serial dilutions of CDX-527. Green lines are association and dissociation curves, red lines represent model fit.

.

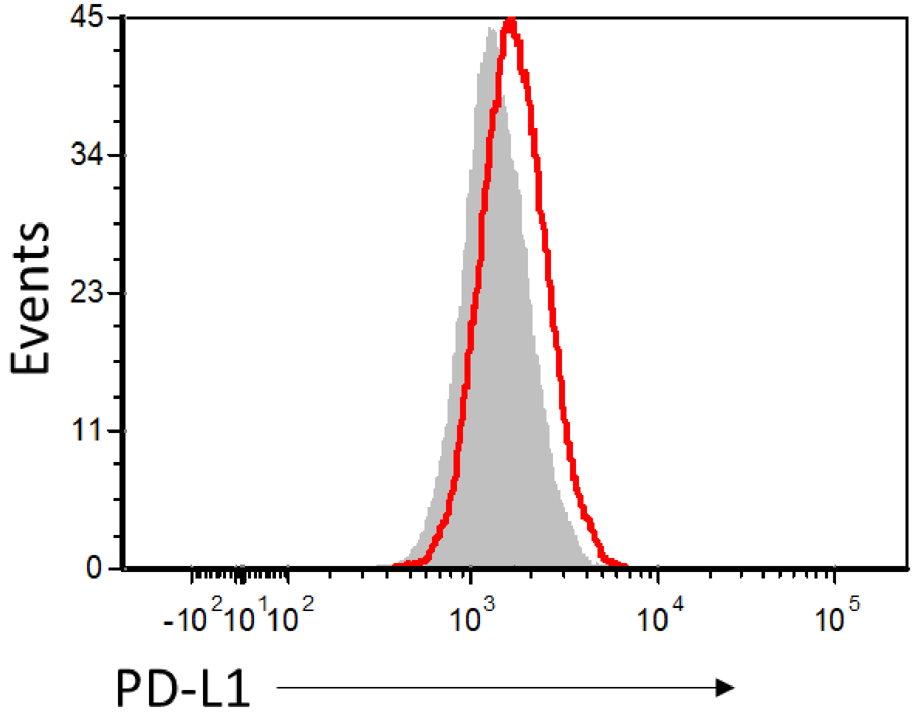

Supplementary Fig. 2. HEK293 reporter cell expression of PD-L1. NFκB reporter HEK293 cells expressing CD27 were incubated with a PE-labeled anti-PD-L1 antibody (red line) or isotype control (gray filled) and analyzed on a FACSCanto II™ instrument.

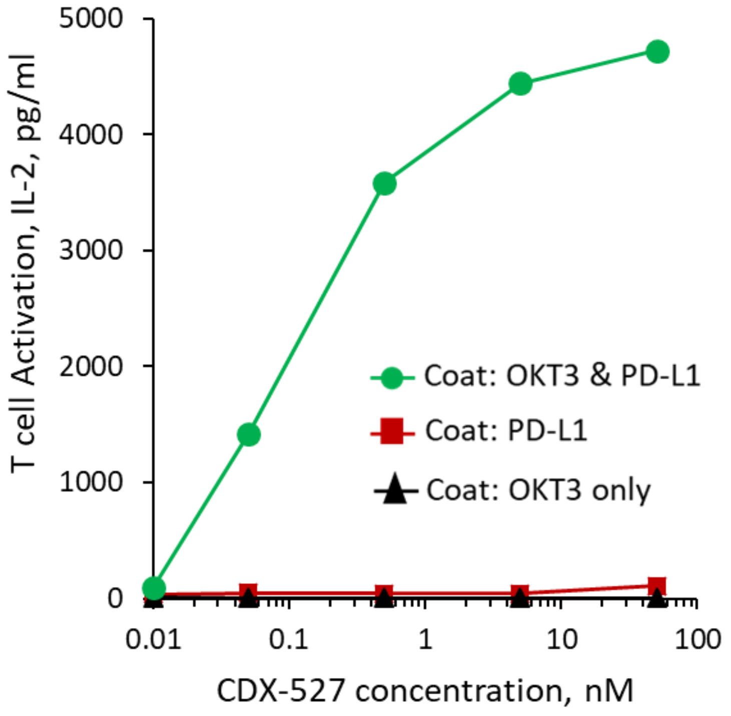

Supplementary Fig. 3. CDX-527 costimulation of T cells requires simultaneous TCR stimulation and cross-linking. Primary human T cells and CDX-527 were added to a plate previously coated with suboptimal OKT3, PD-L1, or both as indicated. IL-2 production in the supernatant by T cells was analyzed by ELISA.

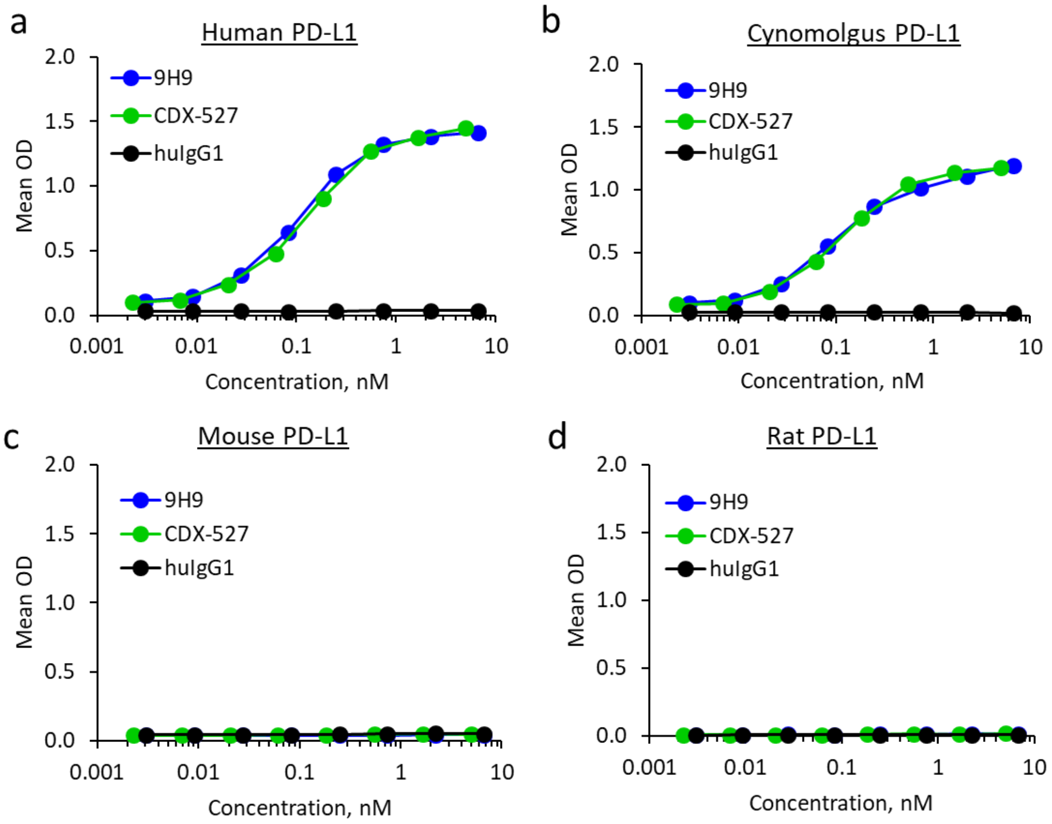

Supplementary Fig. 4. Species reactivity of CDX-527 and mAb 9H9 with PD-L1. Species specific PD-L1-huFc (as indicated in **a-d**) were coated at 2 μg/mL. Samples were added for 1 hour and binding was detected using goat anti-huF(ab')$_2$ HRP.
